# Perceptions of ChatGPT- and veterinarian-generated responses to pet owners’ medical questions

**DOI:** 10.3389/fvets.2026.1939644

**Published:** 2026-10-02

**Authors:** Aïnoha Griot, Rob Ward

**Affiliations:** Department of Veterinary Medicine, The Royal School of Veterinary Studies, University of Edinburgh, Edinburgh, United Kingdom

**Keywords:** artificial intelligence–AI, ChatGPT, client perception, companion animal medicine, digital health, trust, veterinary communication, veterinary medicine

## Abstract

**Background:**

Artificial Intelligence (AI) is becoming increasingly significant in veterinary medicine, particularly in answering pet owners’ medical questions. AI’s integration could reduce veterinary professionals’ workload while improving access to medical advice. However, the reliability and quality of AI-generated responses compared to veterinarians’ remain unexplored. The objective was to determine whether AI-generated responses were perceived as comparable to veterinarian-generated responses.

**Methods:**

This cross-sectional study analysed the answers to a sample of pet owners’ medical questions. Both a veterinarian and ChatGPT (GPT-4o, May 2024) provided answers, which were anonymised and presented in a survey distributed through mailing lists and public Facebook groups. Participants were asked to assess each response based on structure, quality, and empathy, using a five-point Likert scale. They then had to indicate whether they trusted the response, and which response they preferred.

**Results:**

Over two-thirds of the 201 participants (68%) preferred the ChatGPT-generated answers (*p* < 0.05), which were rated higher in structure, quality, and empathy.

**Limitations:**

This study has several limitations, including a small sample size, reliance on a single veterinarian, and use of only two Reddit-derived scenarios. Response accuracy was not assessed, and word count restrictions may have affected perceived response quality. Additionally, prior research suggests that irrelevant patient details can impact ChatGPT’s accuracy, raising questions about user trust if clinical inaccuracies were present.

**Conclusion:**

AI-generated responses received higher participant ratings in structure, quality and empathy, suggesting AI may enhance veterinary client communication when used under appropriate professional supervision.

## 1 Introduction

Advances in Artificial Intelligence (AI), particularly with Large Language Models (LLMs) such as ChatGPT (Generative Pre-trained Transformer), have gained significant attention since its launch in November 2022 (1), including in veterinary medicine (2). Chatbots’ ability to generate human-like responses to written enquiries makes them highly accessible to a wide audience, providing clear, concise information while also producing detailed and technical advice (3). ChatGPT is among the fastest-growing applications in history, reaching 100 million users within only 2 months of its release (4).

ChatGPT has shown considerable potential in clinical settings, assisting with triage, history-taking, summarising clinical records and generating SOAP notes. These capabilities are increasingly being incorporated into AI-powered veterinary scribes, which automatically transcribe consultations and generate structured clinical documentation, reducing administrative burden and allowing veterinarians to devote more time to patient care and client communication. In veterinary education, the recently introduced VetClinPathGPT acts as a virtual tutor by providing information exclusively from authorised textbooks and the eClinPath website (5). In research, AI can assist with summarising scientific literature, identifying AI-generated text, and accelerating the development of new drugs and treatments (6). AI has also demonstrated promise in diagnostic support. For example, ChatGPT-generated simplified radiology reports were found to be generally factually accurate and clinically useful, although occasional errors and omissions were identified following specialist review (7). Similarly, a deep learning algorithm demonstrated a high degree of agreement with board-certified veterinary radiologists when measuring vertebral heart scores (VHS) on canine and feline radiographs (8). More recently, ChatGPT has also been evaluated as a tool for generating client education handouts for common veterinary internal medicine conditions. The AI-generated handouts were considered accurate and clinically appropriate following specialist review and significantly improved pet owners’ understanding of diseases such as diabetes mellitus, immune-mediated haemolytic anaemia and inflammatory bowel disease, highlighting the potential of generative AI to support client communication (9).

However, concerns remain regarding the reliability of AI in medical applications, particularly in diagnosis. A 2024 study found that ChatGPT answered only 49% of 150 Medscape clinical questions correctly (10). Furthermore, “artificial hallucinations,” whereby AI generates plausible but incorrect information, are a well-documented limitation. These errors may arise because LLMs generate responses based on statistical patterns that can incorporate inaccurate or biased information (11). In veterinary medicine, ChatGPT struggled to diagnose paroxysmal dyskinesia when presented with canine clinical cases from the Royal Veterinary College (12). It has also been shown to have limitations in clinical reasoning, may produce incorrect diagnoses when presented with irrelevant information, and can generate hallucinated or unsupported diagnostic suggestions (13–15). These limitations highlight the importance of using AI cautiously in healthcare settings where inaccurate advice may affect patient outcomes.

Beyond direct clinical use, AI is also being explored in veterinary education and scientific writing, from AI-supported case-based learning to concerns about distinguishing AI-generated text from that of students and researchers (16–20). These developments have highlighted the need for appropriate governance and professional guidance regarding the use of AI in veterinary medicine. Since this study was conducted, the Royal College of Veterinary Surgeons (RCVS) has published guidance on the responsible use of AI in veterinary practice, emphasising that AI should support, rather than replace, veterinary professionals, while clinicians remain accountable for decisions made using AI-assisted tools (21). More broadly, the European Union’s AI Act has introduced the first comprehensive legal framework for AI, reflecting the growing importance of safe, transparent and risk-based AI deployment across healthcare and related professions (22).

Despite this clinical potential and these well-documented limitations, no study to date has directly compared how pet owners themselves perceive AI-generated versus veterinarian-generated answers to their own real-world medical questions, in terms of communication quality and empathy. This is an increasingly important gap, given the widespread availability of online veterinary forums such as Reddit/AskVet for veterinary advice (23). With increasing reliance on online platforms and AI for veterinary advice, this study aimed to evaluate whether ChatGPT’s responses to pet owners’ medical questions were comparable with those of a veterinarian in terms of quality, empathy and structure. By examining how AI-generated advice is perceived by veterinary professionals, students and the public, this study aims to provide insights into both the opportunities and the limitations of AI-assisted communication in veterinary medicine.

## 2 Materials and methods

The survey was approved by the University of Edinburgh Human Ethical Review Committee and the first page of the survey included a statement of consent. A full copy of the final survey can be found in Appendix 1 (Supplementary Materials).

Two scenarios were randomly selected from Reddit/AskVet, a public forum with 318,000 members seeking veterinary advice. Only two scenarios were selected to minimise survey fatigue and maximise completion rates. A UK-based licensed mixed-practice veterinarian with 25 years of clinical experience, who was actively practising and also involved in veterinary teaching, was asked to respond to each scenario in 150–250 words without knowledge of the study’s purpose. The veterinarian had some prior experience answering pet owners’ questions on online forums, although this was not a regular part of their professional activities. ChatGPT (GPT-4o, May 2024) was given the same prompt in a new session, with no prior context. The word limit was imposed to make the responses more comparable and reduce obvious differences in response length between the veterinarian and ChatGPT.

Responses were anonymised as “Answer 1” and “Answer 2” and randomised for each scenario. To prevent bias, the survey title was changed to exclude any AI mention.

The survey was distributed via mailing lists at the RDSVS and UK veterinary Facebook groups. Participants identified their profession or background, rated responses on structure, quality, and empathy using a five-point Likert scale, and indicated which answer they preferred. The quality ratings reflected participants’ subjective perceptions of the responses rather than an objective assessment of clinical accuracy. This process was repeated for both scenarios. On the final page, participants rated statements on AI’s role in veterinary medicine. Participants could not return to previous pages in the survey, ensuring that their responses remained unchanged.

Data was exported to Microsoft Office Excel 2025 (Version 16.94) and analysed using Excel and Minitab 22. A workflow summary is provided in Figure 1.

**Figure 1:**
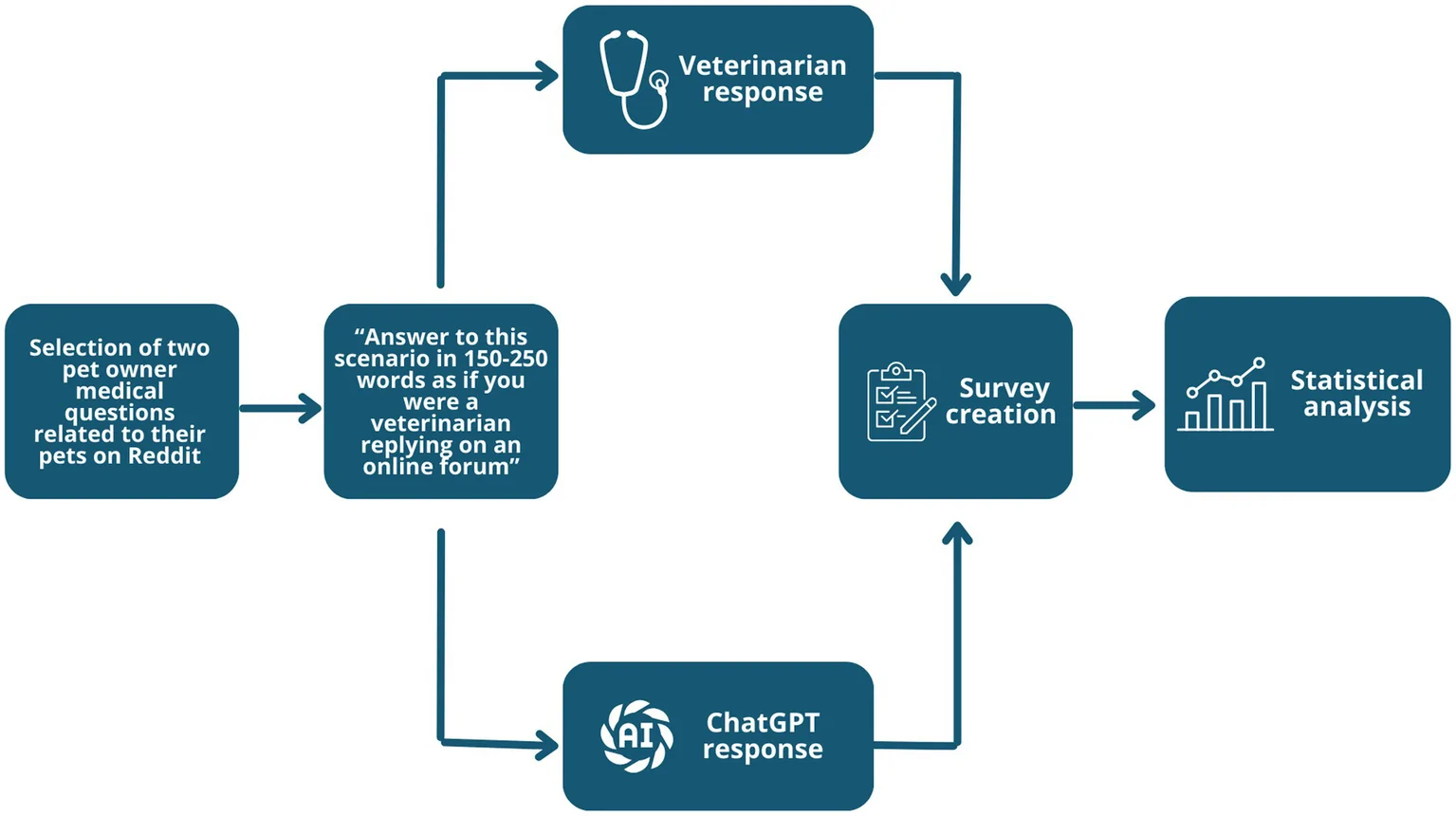
Workflow summary. After selecting two questions on Reddit/AskVet, a veterinarian and ChatGPT were asked to answer to both scenarios; the structure, quality and empathy were assessed in a survey; the results were then analysed.

## 3 Results

Of the 201 participants, there were 43 veterinarians, 22 pre-clinical vet students, 48 clinical vet students, 10 vet nurses, five vet receptionists, 15 researchers or in the medical field, and 58 were members of the public.

### 3.1 Preferred answer and trust in the information provided

Overall, 68.1% (95% CI, 61.7–74.6%) of participants preferred the answer generated by ChatGPT across both scenarios (*p* < 0.05) (Figure 2). Furthermore, 88% (95% CI; 83.3–92.3%) trusted ChatGPT’s answers, whereas only 80% (95% CI; 74.3–85.4) trusted the veterinarian’s answer (Figure 3).

**Figure 2:**
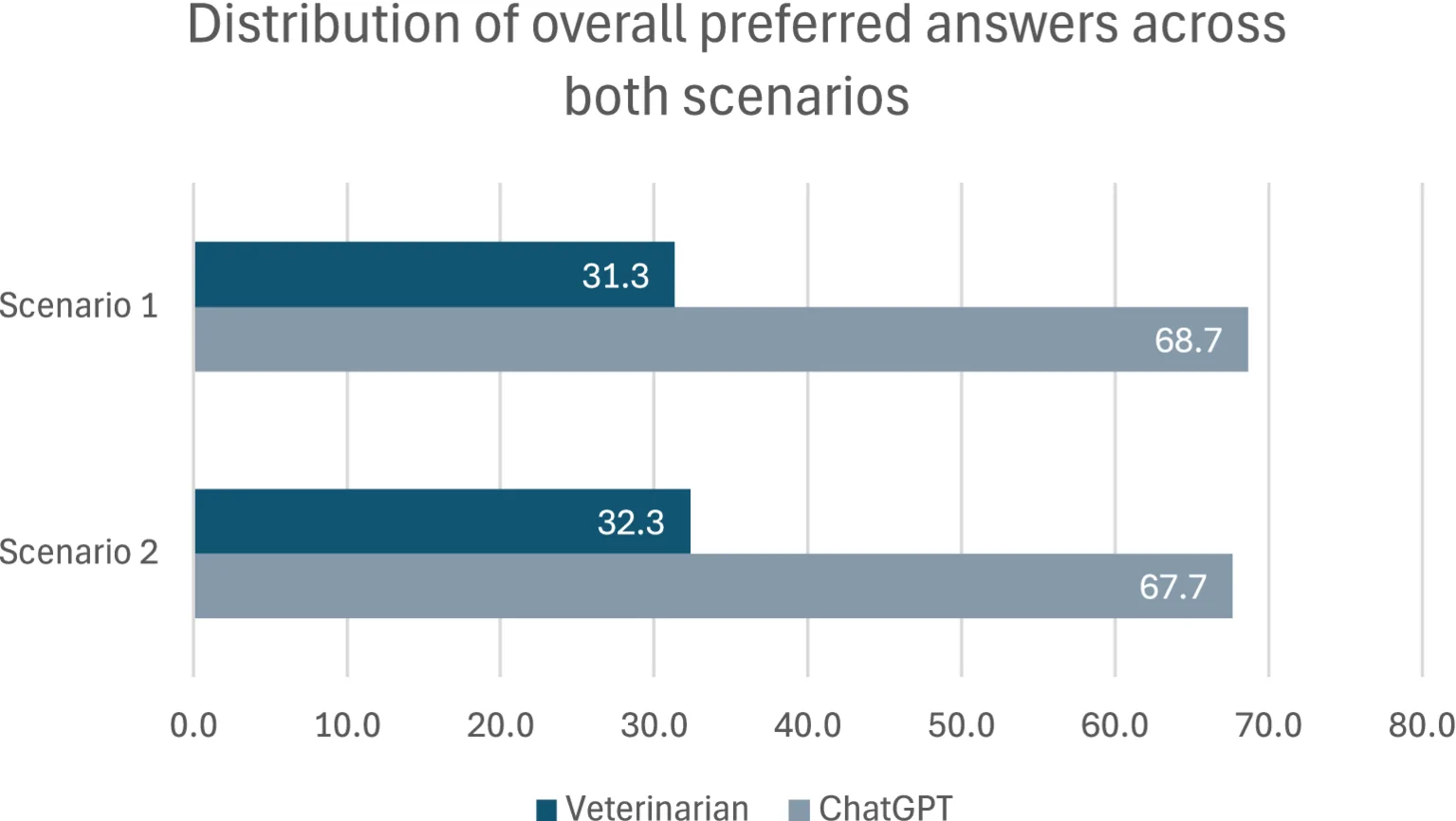
Bar charts representing the proportion of overall preferred answers across both scenarios (in percentage).

**Figure 3:**
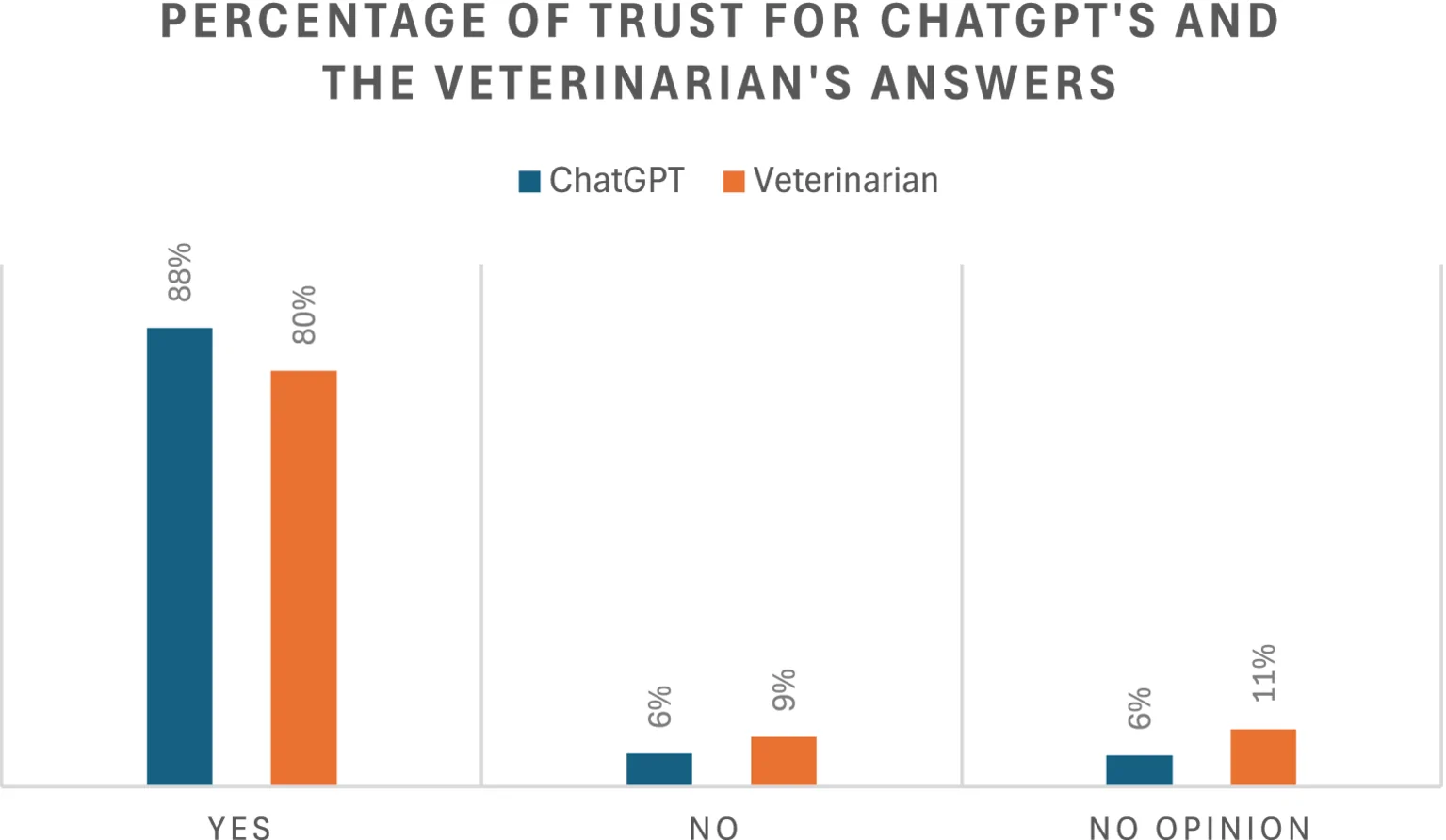
Bar chart representing the proportion of participants who trusted ChatGPT’s and the veterinarian’s answers across both scenarios.

### 3.2 Likert scale analysis

To assess differences in participant ratings between ChatGPT and the veterinarian, mean scores for structure, quality, and empathy were calculated (Figure 4), and paired t-tests were conducted.

**Figure 4:**
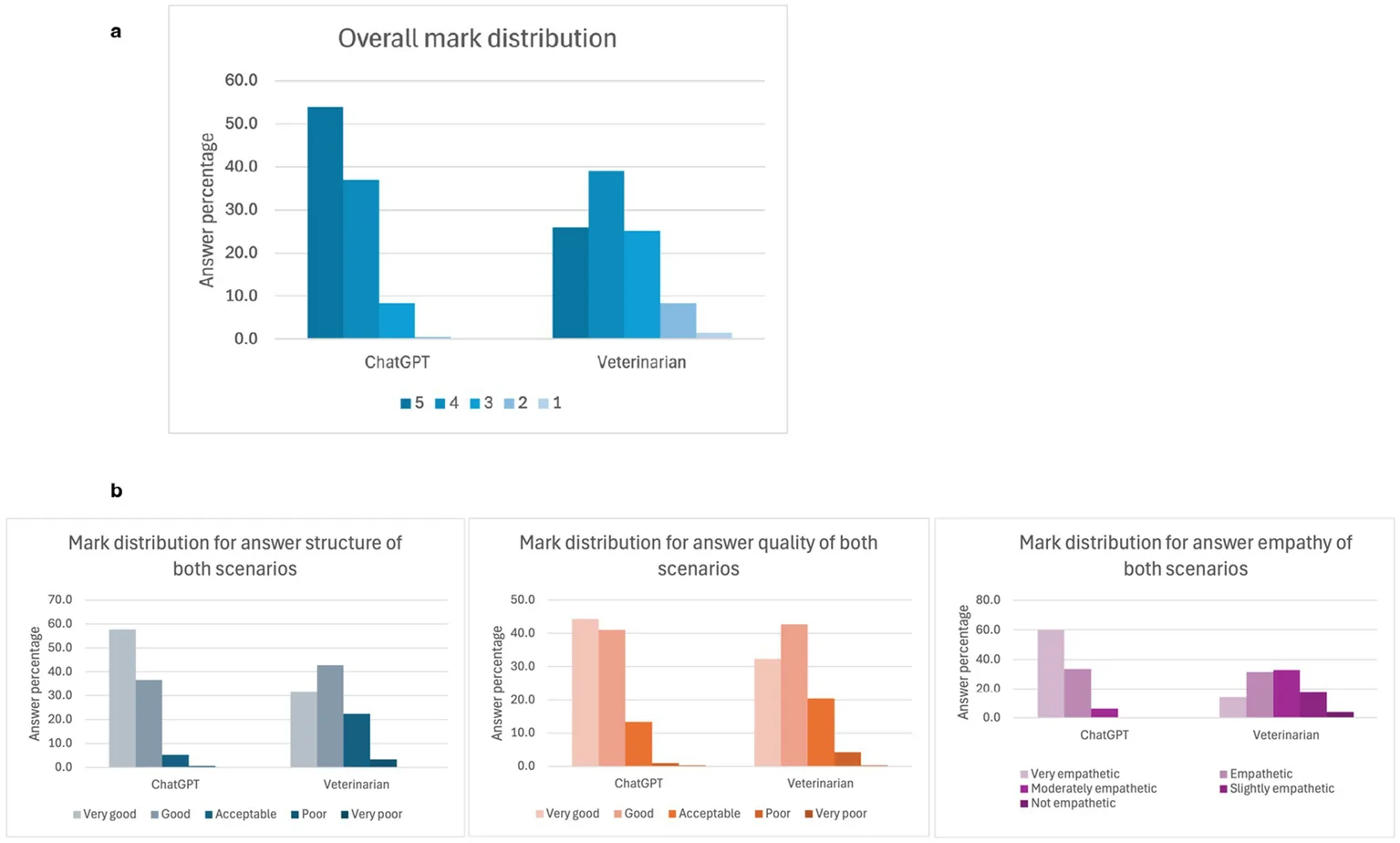
Bar charts showing the mark distribution across both scenarios (in percentage): **(a)** Overall mark distribution for ChatGPT’s and the veterinarian’s answers. **(b)** Mark distribution for the structure, quality, and empathy.

#### 3.2.1 Structure

Participants rated ChatGPT’s structure significantly higher than the veterinarian’s (t = 10.78; *p* < 0.05), with a mean score of 4.52 (95% CI, 4.45–4.58) compared to 4.03 (95% CI, 3.94–4.11).

ChatGPT’s responses were rated as good or very good (>3) 94.3% of the time (95% CI, 91.1–97.5%), compared to 74.4% (95% CI, 68.3–80.4%) for the veterinarian—a 1.27 times higher prevalence. Only two participants rated ChatGPT’s structure as poor (<3), compared to 13 for the veterinarian, a 6.5-fold difference.

#### 3.2.2 Quality

In terms of the quality of the information provided by each answer, the difference in ratings between ChatGPT’s and the veterinarian’s responses was once again significant (t = 4.64; *p* < 0.05). The mean score for the quality of ChatGPT’s responses was 4.28 (95% CI, 4.19–4.37), while the veterinarian’s responses had a mean score of 4.03 (95% CI, 3.93–4.12).

The veterinarian’s answers had 3.6 times more “poor” or “very poor” answers compared to ChatGPT – 18 responses for the veterinarian, five for ChatGPT.

The proportion of responses rated as good or very good (>3) was not significantly different between the veterinarian (75.1% with 95% CI, 69.1–81.1%) and ChatGPT (85.3% with 95% CI, 80.4–90.2%).

#### 3.2.3 Empathy

The largest difference in rating was in empathy ratings (t = 20.12; *p* < 0.05). The mean score for the empathy of ChatGPT’s responses was 4.53 (95% CI, 4.46–4.60), while the veterinarian’s responses had a significantly lower mean score of 3.35 (95% CI, 3.23–3.46).

Moreover, a 2.04 times higher prevalence of responses rated good or very good (>3) was noted for the empathy ratings of ChatGPT: this prevalence was of 93.6% for ChatGPT (95% CI, 90.1–96.9%), and only 45.8% for the veterinarian (95% CI, 38.9–52.7%).

Regarding the proportion of responses rated as not empathetic or slightly empathetic (<3), ChatGPT was rated as such in only 0.2% of cases (95% CI, 0–0.94%), whereas this amounted to 21.4% (95% CI, 15.7–27.1%) of the veterinarian’s ratings.

### 3.3 Differences in ratings depending on background

To examine whether the general public and participants with a veterinary background rated the responses differently, a series of ANOVA (Analysis of Variance) tests and paired t-tests were conducted.

The “Public” included all the participants who identified as “I am a member of the general public” (n = 58), whereas “Participants with Veterinary Background” included veterinarians (n = 43) and vet students (n = 60). Other categories were excluded from this last group due to small sample sizes, or because of the differences in clinical reasoning trainings.

First, a one-way ANOVA was performed for both the veterinarian’s and ChatGPT’s answers combined, and showed that the public rated the structure of answers higher than participants with a veterinary background (*F* = 4.70; *p* < 0.05). The public also rated quality significantly higher than veterinary professionals (*F* = 12.87, p < 0.05). However, there was no significant difference in the ratings of empathy between the two groups (*F* = 2.36, *p* > 0.05).

To determine whether these differences persisted when considering the answers from ChatGPT and the veterinarian separately, two separate sets of ANOVA tests were conducted, one for ChatGPT’s answers, and one the veterinarian’s answers.

For ChatGPT’s answers, the public rated both the structure (*F* = 4.43, *p* < 0.05) and the quality (*F* = 18.93, *p* < 0.05) significantly higher than veterinary professionals. However, there was no significant difference for the veterinarian’s responses (Structure: *F* = 2.06, *p* > 0.05; Quality: *F* = 1.91, *p* > 0.05).

### 3.4 General questions on artificial intelligence

Overall, 65% (95% CI; 58.1–71.3%) of participants agreed that AI could help a veterinarian reach a diagnosis and a treatment plan, but a strong majority (94% of participants; 95% CI; 91.4–97.7%) would not trust an AI to do so without the assistance of a veterinarian. Although 63% (95% CI; 57.0–70.3%) of participants do believe that AI will have a crucial role in veterinary medicine in the years to come, almost one in five participants do not agree (19% of participants; 95% CI; 13.1–23.8%). A summary of these findings can be found in Figures 5, 6.

**Figure 5:**
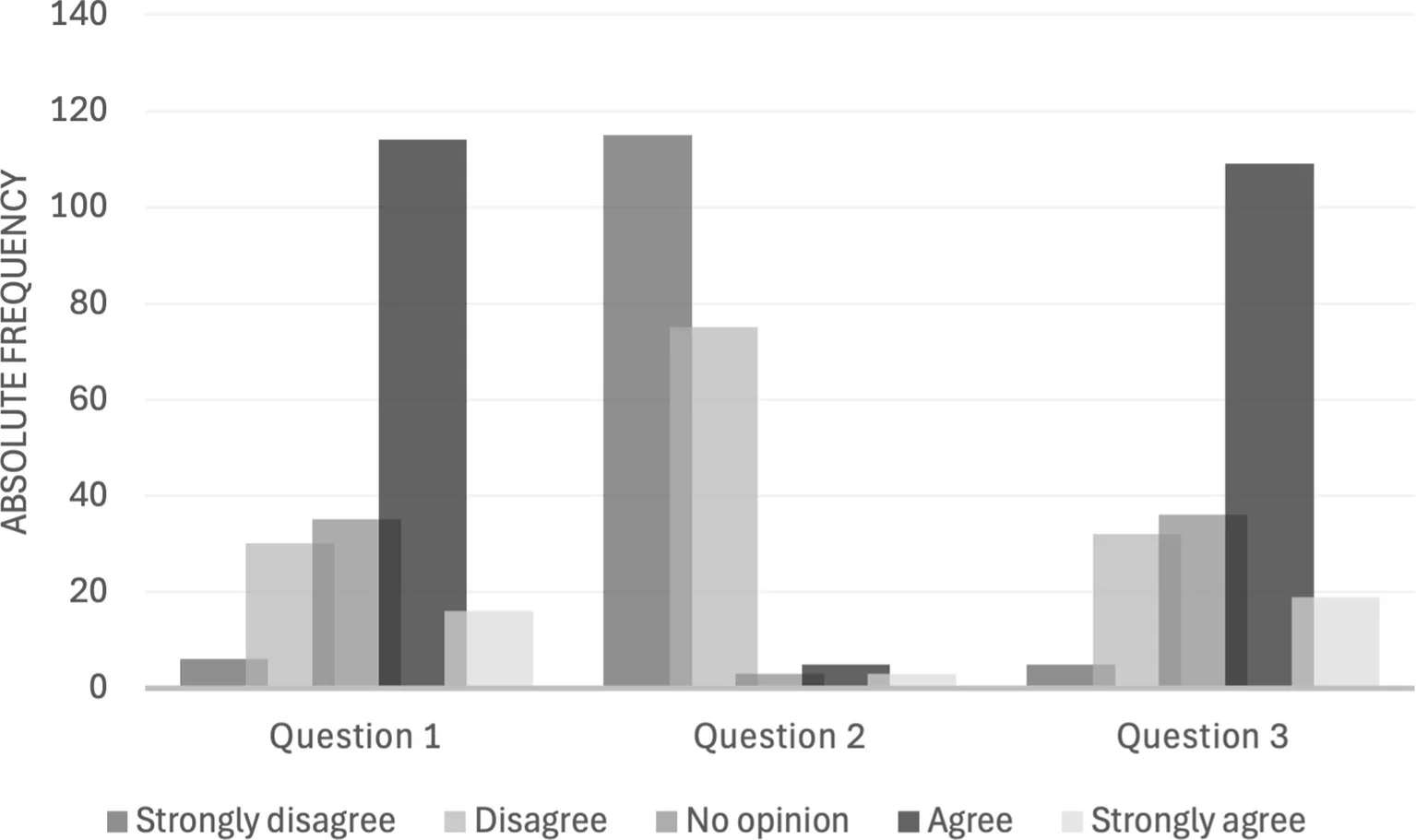
Bar charts representing the frequency of the participants’ ratings for statements related to artificial intelligence. Question 1. Artificial intelligence can help a veterinarian reach a diagnosis and a treatment plan. Question 2. I would trust an artificial intelligence to formulate a diagnosis and a treatment plan for a pet without assistance from a veterinarian. Question 3. Artificial intelligence will have an essential role in veterinary medicine in the years to come.

**Figure 6:**
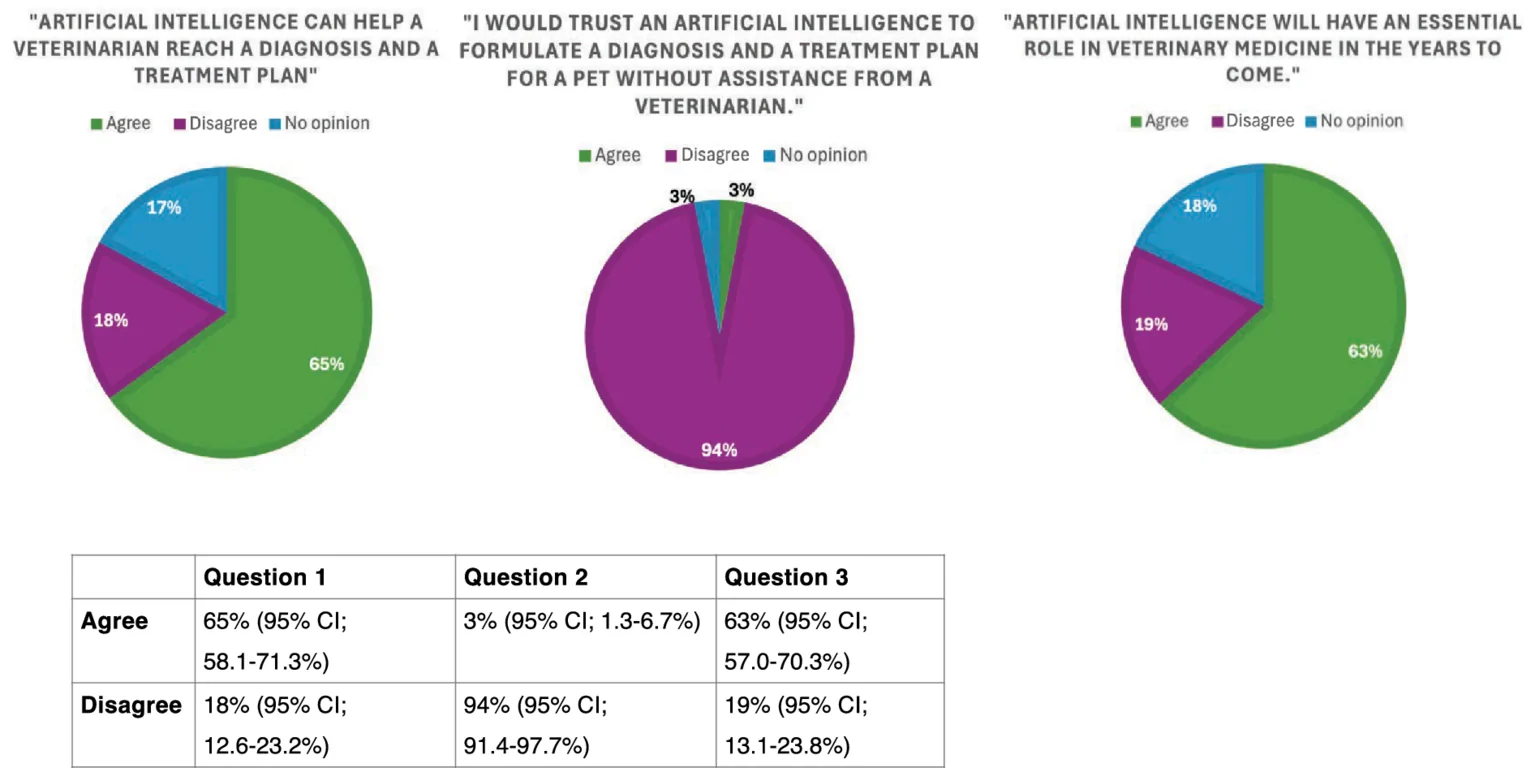
Pie charts representing the extent to which participants agree with statements relating to the role of Artificial Intelligence in veterinary medicine.

## 4 Discussion

In this study, participants rated ChatGPT responses higher for structure, perceived quality and perceived empathy; however, this study deliberately evaluated participant perceptions rather than the clinical accuracy of the responses. A closer examination of the AI-generated responses revealed that ChatGPT consistently included a brief introduction and conclusion and used more empathetic language, whereas the veterinarian’s answers were more factual and direct. The clearer organisation and communication style of the responses may have contributed to participants’ higher ratings for structure and perceived quality.

The most notable difference was observed in perceived empathy, with mean scores of 4.53 for ChatGPT compared with 3.35 for the veterinarian. However, written scenarios cannot fully replicate a real-life veterinary consultation, where verbal communication, body language and in-person interactions all play an important role in establishing trust and demonstrating empathy (24). Furthermore, the scenarios provided only limited clinical information. During a consultation, veterinarians would have access to the patient’s medical history and could ask follow-up questions before providing advice, both of which are essential for accurate clinical decision-making. The limited context available in this study may therefore have contributed to the veterinarian’s shorter and more direct responses.

This finding should not be interpreted as evidence that AI possesses genuine empathy. Rather, it highlights how empathy is expressed in written interactions specifically. ChatGPT’s communication style frequently includes explicit acknowledgement of owners’ concerns, reassuring language and a clear, structured presentation of information - features that have recently been associated with higher perceived empathy ratings in text-based medical interactions (25). Adapting written communication to incorporate these features could improve client satisfaction and trust while encouraging pet owners to continue seeking professional veterinary advice rather than relying solely on AI. Veterinary education may therefore benefit from placing greater emphasis on explicit written expressions of empathy and reassurance, particularly for online communication where non-verbal cues are absent. Rather than viewing AI-generated text as competition, it could be used as a teaching example, helping students identify language that effectively conveys empathy while reinforcing that genuine empathy, ethical reasoning and professional judgement remain fundamental to veterinary practice. These insights could also inform both undergraduate teaching and continuing professional development, helping veterinarians deliver advice that is both clinically accurate and perceived as more supportive.

Nevertheless, perceived empathy is likely to depend on the communication context, and AI’s apparent advantage in this study should not be generalised across all veterinary communication tasks. In a recent comparison of ChatGPT- and veterinary student-generated discharge handouts for canine neurological conditions, veterinary educators rated the student-written handouts as significantly more empathetic and supportive of clients than those produced by ChatGPT, suggesting that AI’s communication advantages may not extend uniformly across all veterinary tasks and audiences (26). Rather than demonstrating an inherent advantage of AI, these findings indicate that perceived empathy is influenced by the specific communication task, the comparison group and the design of the language model. They reinforce the importance of evaluating AI performance within the context in which it is intended to be used.

Participants with a veterinary background were generally more critical than members of the public when evaluating ChatGPT’s responses, particularly regarding structure and quality. Compared with the veterinarian’s responses, ChatGPT occasionally used less medically specific and precise terminology, which may have been more readily recognised by participants with veterinary training. This finding suggests that clinical expertise influences how AI-generated information is evaluated and highlights the importance of involving veterinary professionals in the assessment and implementation of AI tools.

Furthermore, more than two-thirds of participants preferred the response generated by ChatGPT, suggesting that empathy and writing style may contribute to perceptions of credibility and overall preference. As digital communication increasingly forms part of veterinary practice (27), this preference may encourage greater reliance on AI tools alongside traditional veterinary services. Consequently, veterinarians may face increasing expectations regarding the clarity, empathy and accessibility of their written communication. Conversely, AI may provide an opportunity to improve these aspects of client communication by demonstrating approaches that are consistently perceived as supportive and easy to understand.

This apparent preference for, and trust in, ChatGPT should be considered alongside participants’ broader views on AI’s clinical role. Although 88% of participants reported trusting the information provided by ChatGPT, compared with 80% for the veterinarian, most indicated that they would not rely on AI alone to generate a diagnosis or treatment plan. Nevertheless, nearly two-thirds believed AI could assist veterinarians and would play an important role in the future of veterinary care. Taken together, these findings suggest broad acceptance of AI as a clinical support tool rather than an autonomous decision-maker. Human interaction, ethical reasoning and professional judgement provided by veterinary professionals remain fundamental to veterinary practice (15). As Dr. Richard Goldstein, Zoetis’ Global Chief Medical Officer, stated regarding Vetscan Imagyst, an AI-powered diagnostic platform, “the idea is not to replace a person, but enhance what a person can do” (28).

This distinction between support and replacement is particularly important given that AI-generated responses remain susceptible to misinformation and “artificial hallucinations” (11). Confidently presented but inaccurate advice could lead pet owners to make inappropriate decisions regarding their animals’ care. The need for professional oversight is reinforced by recent veterinary research: an external validation of six commercially available veterinary radiology AI services found variable diagnostic performance and identified important deficiencies in image interpretation, highlighting the need for continued validation and clinician oversight before such systems are relied upon in clinical practice (29). When used under appropriate professional supervision, however, AI could enhance communication with clients while maintaining the safety and accuracy of the advice provided (22, 30).

This is consistent with recent guidance published by the Royal College of Veterinary Surgeons (RCVS), which recommends that AI should support, rather than replace, veterinary professionals, while emphasising that clinicians remain responsible for evaluating AI-generated outputs and for all clinical decisions (21). In addition to the RCVS guidance, the UK Information Commissioner’s Office has published recommendations on the responsible deployment of AI, highlighting the importance of transparency, accountability and the protection of personal data when AI systems are used in professional settings (30). Together with broader regulatory developments such as the European Union’s AI Act (22), these frameworks reinforce that, as AI becomes increasingly integrated into veterinary practice, it should complement clinical practice while remaining subject to appropriate professional oversight and regulatory safeguards.

### 4.1 Limitations

One of the key limitations of this study is the relatively small sample size, which limits the generalisability of the findings. In addition, only two Reddit scenarios were included to minimise survey fatigue, maximise survey completion and reduce participant dropout. While this approach likely improved participation, it limited the range of clinical presentations evaluated. Future studies should include a broader variety of cases requiring different levels of clinical reasoning to determine whether these findings remain consistent across more diverse scenarios. Furthermore, only one veterinarian contributed responses to the study. Although they had 25 years of clinical experience in UK mixed practice and were actively involved in both clinical work and veterinary teaching, their communication style may not be representative of the wider veterinary profession. Consequently, the findings should not be interpreted as representative of veterinarians as a whole.

Another important limitation is that the clinical accuracy of the responses was not independently evaluated. Consequently, it was not possible to determine whether higher participant ratings reflected superior communication alone, clinically accurate advice, or a combination of both. Patient histories frequently contain clinically irrelevant information, and previous research suggests that such information can reduce ChatGPT’s diagnostic accuracy when generating medical advice (14). If ChatGPT’s responses contained clinical inaccuracies, it would be valuable to investigate whether participants would continue to prefer and trust them to the same extent, and whether these perceptions differ between individuals with and without veterinary training.

As AI systems have evolved rapidly since data collection, both the capabilities of ChatGPT and the professional guidance surrounding AI have changed. Consequently, the findings should be interpreted within the context of the version of ChatGPT (GPT-4o, May 2024) evaluated in this study. In addition, responses generated by large language models are sensitive to prompt wording. Although ChatGPT received the same prompt as the veterinarian and each response was generated in a new conversation, alternative prompts or prompting strategies may have produced different responses in terms of structure, tone or content. Accordingly, these findings reflect ChatGPT’s performance under the specific prompting conditions used in this study rather than its overall capabilities.

The imposed word limit may also have influenced the natural communication style and depth of the responses provided by both ChatGPT and the veterinarian. Although the same word limit was applied to improve comparability between responses, it remains unclear whether differences in response length under routine conditions would influence participant ratings. In a comparable study in human medicine, physicians’ responses were significantly shorter and less detailed than those generated by an AI chatbot, suggesting that response length itself may contribute to perceptions of communication quality (31).

Future research should therefore evaluate a larger and more diverse range of veterinary scenarios involving multiple veterinarians, independently assess the clinical accuracy of AI-generated responses, and investigate whether the communication advantages observed in this study persist across newer language models and alternative prompting strategies.

## 5 Conclusion

Overall, this study highlights the considerable potential of AI to support veterinary communication. Participants consistently rated the AI-generated responses more favourably than those of the veterinarian included in this study in terms of structure, perceived quality and perceived empathy. In a profession where veterinary teams are known to experience high workloads (32), AI may help reduce administrative workload by assisting with client communication, drafting written advice and supporting clinical documentation.

These findings should, however, be interpreted alongside the evolving regulatory and professional framework for AI in veterinary medicine, including recent guidance from the Royal College of Veterinary Surgeons and the European Union’s AI Act, both of which emphasise that AI should support, rather than replace, veterinary professionals, with clinicians remaining accountable for all clinical decisions (21, 22).

Beyond governance, this study highlights a more immediate opportunity for the profession. Much of ChatGPT’s advantage appeared to lie not in demonstrating superior clinical expertise, but in communicating information through clear structure, explicit acknowledgement of owners’ concerns and reassuring language. These are teachable communication skills rather than capabilities unique to AI. Incorporating greater emphasis on written empathetic communication into veterinary education and continuing professional development could help improve client satisfaction while maintaining the central role of professional clinical judgement. Ultimately, AI should be viewed as a complementary tool that can enhance communication and efficiency, provided its outputs are critically evaluated and veterinary professionals remain responsible for patient care.

## Data Availability

The original contributions presented in the study are included in the article/Supplementary material, further inquiries can be directed to the corresponding author.
