# Supplementary material for: Perceptions of ChatGPT- and veterinarian-generated responses to pet owners’ medical questions
Source: Front Vet Sci. 2026 Oct 2;13:1939644. doi: 10.3389/fvets.2026.1939644 (PMC13672839; doi:10.3389/fvets.2026.1939644)
Supplement: Supplementary file 1 [file Supplementary_file_1.DOCX]

APPENDICES

Appendix 1: Questionnaire sent out via mailing lists and posted on Facebook groups.

*Note (not included in the survey): Answer 1 for Scenario 1 and Answer 2 for Scenario 2 were generated by ChatGPT*

**Page One**

First of all, please select which option applies to you:

- I am a veterinarian
- I am a vet student (pre-clinical year)
- I am a vet student (clinical year)
- I am a vet nurse
- I am a vet receptionist/ animal care assistant
- I have medical background (researcher, other medical studies...)
- Member of the general public

**Page Two: First Scenario**

I rescued a 5-month-old Beagle. She is the sweetest puppy, but when she walks, she is not able to stand normally on her back legs – they are very turned out. She does not have incontinence*. Do you have any idea of what the issue may be?

*she is able to pass urine and faeces normally

**Page 3: Answer 1**

It’s heartwarming to hear that you rescued a Beagle puppy; she’s lucky to have such a caring owner! I’m sorry to hear that she’s having trouble standing properly on her back legs. This could be related to a few different conditions.

One possibility is hip dysplasia, a common condition in some dog breeds, including Beagles, where the hip joint doesn't develop properly. This can lead to abnormal walking patterns. Another potential issue could be related to her growth plates or limb alignment. If her legs are rotated outward, it may indicate a problem with her joints, muscles, or bones, which could affect her gait.

In some cases, puppies may have developmental issues that cause temporary gait abnormalities, but if this is persistent, it's worth consulting a vet. They can perform a thorough examination, including X-rays if necessary, to determine if there’s any structural problem or discomfort. Given that she does not have incontinence or other neurological signs, it's less likely to be a spinal issue, but a vet will be able to rule out any concerns.

Your puppy’s comfort and well-being are important, and with the right care, she can still live a happy, active life. Keep her on a balanced diet and avoid strenuous exercise until you have more information.

Based on this answer, how would you rate:

The structure of the response:

- Very poor
- Poor
- Acceptable
- Good
- Very good

The quality of information provided:

- Very poor
- Poor
- Acceptable
- Good
- Very good

The empathy demonstrated:

- Not empathetic
- Slightly empathetic
- Moderately empathetic
- Empathetic
- Very empathetic

Do you trust the information provided?

- Yes
- No
- No opinion

**Page 4: Answer 2**

Congratulations on your new puppy! The beagle is what we call a “chondrodystrophic” breed – the term refers to abnormalities in the development of cartilage (including the growth points of the limb bones). Most of these dogs have the “short leg long body” shape of e.g. dachshunds, but their legs are often quite curved / bowed, which can be “normal” for that dog. It would depend upon what you mean by “not able to stand normally” (can she stand up at all?), the degree to which the limbs are turned out and how this affects her day-to-day.

She’s a puppy, so this could represent something congenital (i.e. she was born this way) - but as she’s a rescue, there could be other historical things that we don’t know about. There’s a long list of potential issues – from previous trauma / musculoskeletal injury, to something developmental / congenital, nutritional, neurological, cardiovascular etc. I’d suggest taking her to have her examined by your vet as they will be able to help you rule in/out some of these possibilities and advise you accordingly.

Based on this answer, how would you rate:

The structure of the response:

- Very poor
- Poor
- Acceptable
- Good
- Very good

The quality of information provided:

- Very poor
- Poor
- Acceptable
- Good
- Very good

The empathy demonstrated:

- Not empathetic
- Slightly empathetic
- Moderately empathetic
- Empathetic
- Very empathetic

Do you trust the information provided?

- Yes
- No
- No opinion

**Page 5**

Overall, which answer did you prefer?

- Answer 1
- Answer 2

**Page 6: Second (and last!) scenario**

I have a concern regarding my cat. I have just moved to a different country, and finding a vet who speaks English is a bit complicated.

My cat is a three-year-old female, weighs roughly 5kg, and is an indoor cat (aside from escaping a couple of times). She has episodes where it looks like she's sneezing… sort of. She sticks her head out and makes weird wheezing noises, with her head jerking back every time. She has had these episodes since she was a kitten. This is my first cat, and some people tell me it's normal for cats to sneeze, but I am very concerned. I don't know if it's a hairball, asthma, or something else. I'd appreciate any help or advice.

**Page 7: Answer 1**

It is normal for cats to sneeze, though it can be difficult to differentiate this from other things including, as you say, hairballs and asthmatic coughing and wheezing, particularly if you’re not familiar with them. Cats can make pretty weird noises when they’re bringing up hairballs (which aren’t uncommon) but there’s often a lot more effort coming from their tummy than is the case when they’re sneezing or wheezing – plus of course the hairball itself…! That said, cats can have more than one thing “going on” at a time.

The best thing to do would be to have her seen by a vet, who can check her over. They may need a little more detail as to how often these episodes are happening, under what circumstances and for how long each time (so it might be useful to write those down and/or translate them in advance so that everybody understands). I’d suggest that you take a video of one of her episodes, if possible, to show to them.

A vet can do a full examination that might give additional context / clues (in particular, examining her respiratory tract and her breathing as well as feeling her tummy) and help to determine the best course of action.

Based on this answer, how would you rate:

The structure of the response:

- Very poor
- Poor
- Acceptable
- Good
- Very good

The quality of information provided:

- Very poor
- Poor
- Acceptable
- Good
- Very good

The empathy demonstrated:

- Not empathetic
- Slightly empathetic
- Moderately empathetic
- Empathetic
- Very empathetic

Do you trust the information provided?

- Yes
- No
- No opinion

**Page 8: Answer 2**

I completely understand why you’d feel worried, especially being in a new country where language barriers make finding a vet more challenging. What you’re describing — the wheezing, head jerking, and sticking her head out — could be something as simple as reverse sneezing. This can be unsettling to watch, but it’s usually harmless and caused by nasal irritation. It tends to resolve on its own without lasting issues.

Another common cause could be hairballs. Since your cat is an indoor cat and likely grooms frequently, it’s possible she’s trying to expel a hairball. The head movements and sounds you described fit with what cats often do when they’re struggling with hair buildup.

Asthma is also worth considering, although it usually presents with more consistent symptoms like coughing and noticeable difficulty breathing. Since your cat has had these episodes since she was a kitten and they don’t seem to be getting worse, a chronic respiratory condition is less likely but not impossible.

Since communicating with a vet might be tricky, try using translation apps or bringing a video of the episodes for them to see. This can make it easier for the vet to understand what’s happening. If you’re still concerned, consulting an online vet could provide more immediate advice. Your cat’s health and your peace of mind are important, so I hope this helps guide you in the right direction to ensure she stays healthy and happy.

Based on this answer, how would you rate:

The structure of the response:

- Very poor
- Poor
- Acceptable
- Good
- Very good

The quality of information provided:

- Very poor
- Poor
- Acceptable
- Good
- Very good

The empathy demonstrated:

- Not empathetic
- Slightly empathetic
- Moderately empathetic
- Empathetic
- Very empathetic

Do you trust the information provided?

- Yes
- No
- No opinion

**Page 9:**

Overall, which answer did you prefer?

- Answer 1
- Answer 2

**Page 10: Finally, here are a few additional questions:**

“Artificial Intelligence can help a veterinarian reach a diagnosis and a treatment plan.” To what extent do you agree with this statement?

- Strongly disagree
- Disagree
- No opinion
- Agree
- Strongly agree

"I would trust an Artificial Intelligence to formulate a diagnosis and a treatment plan for a pet without any assistance from a veterinarian." To what extent do you agree with this statement?

- Strongly disagree
- Disagree
- No opinion
- Agree
- Strongly agree

“Artificial Intelligence will have an essential role in veterinary medicine in the years to come.” To what extent do you agree with this statement?

- Strongly disagree
- Disagree
- No opinion
- Agree
- Strongly agree
